# Genetic Structure of the Root Vole *Microtus oeconomus:* Resistance of the Habitat Specialist to the Natural Fragmentation of Preferred Moist Habitats

**DOI:** 10.3390/genes13030434

**Published:** 2022-02-27

**Authors:** Rafał Łopucki, Iwona Mróz, Zuzanna Nowak-Życzyńska, Magdalena Perlińska-Teresiak, Edyta Owadowska-Cornil, Daniel Klich

**Affiliations:** 1 Centre for Interdisciplinary Research, The John Paul II Catholic University of Lublin, Konstantynów 1J, 20-708 Lublin, Poland; lopucki@kul.pl; 2 Department of Animal Genetics and Conservation, Warsaw University of Life Sciences-SGGW, Ciszewskiego 8, 02-786 Warsaw, Poland; zuzanna_nowak_zyczynska@sggw.pl (Z.N.-Ż.); magdalena_perlinska_teresiak@sggw.edu.pl (M.P.-T.); 3 The John Paul II Catholic University of Lublin, Racławickie 14, 20-950 Lublin, Poland; imroz@kul.pl; 4 Science and Nature Monitoring Department, Kampinoski National Park, Tetmajera 38, 05-080 Izabelin, Poland; edyta.owadowska@neemo.eu

**Keywords:** population, microsatellite, climate change, rodents, wetlands, barriers, gene flow, migration

## Abstract

Climate-related changes have a severe impact on wetland ecosystems and pose a serious challenge for wetland-dependent animals as their preferred habitats decline, lose spatial continuity, and appear as isolated islands in the landscape. In this paper, we studied the effects of long-term habitat changes (drying out and fragmentation of wet non-forest habitats) on the genetic structure of the population of the root vole *Microtus oeconomus*, a species preferring moist habitats. We intended to check what barriers and what distances affected its genetic isolation on a local scale. The study was conducted in the area of Kampinoski National Park in central Poland (Europe). DNA variability of 218 root vole individuals was assessed by genotyping nine microsatellite loci. Despite its spatial fragmentation, the studied population did not seem to be highly structured, and isolation through distance was the main differentiating factor. Even a distance of several kilometres of unfavourable natural habitats and unfavourable terrain did not exclude the exchange of genes between subpopulations. Our results suggest that the genetic effects of the fragmentation of wetlands have been considerably compensated (delayed) due to the migratory abilities of this species. Our study does not provide clear results on the impact of anthropogenic barriers but suggests that such barriers may have a much stronger effect than natural barriers.

## 1. Introduction

Climate-related changes have a severe impact on natural ecosystems, including wetlands. Wetlands have already suffered greatly in recent decades due to widespread drainage and other anthropogenic changes; therefore, they are more susceptible to climate warming [1,2]. As the temperature rises, evapotranspiration increases, the water level drops, wetland habitats dry up, and the plant cover is radically transformed. Additionally, a stressful effect of summer drought on inland wetlands has been observed [2].

Warming-related changes pose a serious challenge for wetland-dependent animals, as their preferred habitats decline, lose spatial continuity, and appear as isolated islands in the landscape [2,3]. In a patchy habitat, the long-term survival of species cannot be ensured without well-working metapopulation dynamics [4]. Fragmentation of the preferred habitat effectively restricts dispersal, resulting in a reduction in the population size and a restriction of gene flow between populations. Small populations deprived of the opportunity of gene exchange suffer from genetic drift and inbreeding leading to a loss of genetic variability, which increases the differentiation between subpopulations and elevates the risk of local extinction [5,6,7]. A real challenge for authorities responsible for conservation is to predict the acceptable loss of wetlands ensuring that the populations of species associated with these habitats remain viable [8].

The negative effects of habitat fragmentation depend on the species biology, especially its behaviour, habitat requirements, and dispersal abilities [7,9]. Small rodents are often used as a model for studying such processes [10,11,12]. In the case of wet non-forest habitats, the root vole *Microtus oeconomus* (Pallas, 1776) (currently called *Alexandromys oeconomus* [13]) has often been used as a model species to analyse the impact of historical postglacial colonisation [14,15,16,17,18]. The root vole is a small Holarctic mammal species preferring moist habitats, e.g., *Phragmitetea, Scheuchzerio-Caricetea,* and *Molinio-Arrhenatheretea*. In Europe, the south-western border of its continuous range runs across the southern part of Poland [19]. Geographically isolated relict populations of the root vole occur in Norway, the Netherlands, Sweden, Finland, Austria, Slovakia, and Hungary [20,21]. It has been shown that this species may be sensitive to drought and the anthropogenic fragmentation of preferred habitats, which may lead to genetic differentiation between local populations [21,22,23]. However, the significance of the combined effect of anthropogenic and natural barriers related to the ongoing climate change on root vole populations has not yet been sufficiently studied.

In order to study the local effects of the drying up, fragmentation, and isolation of wetlands on the genetic structure of species associated with these habitats, it is important to select appropriate study sites and methods. Nowadays, molecular methods are valuable tools for the analysis of the impact of habitat fragmentation on populations. The most widely used molecular markers are microsatellites (short 2–5 base-pair, repeating sequences of DNA) due to their high polymorphism and possibility of amplification from a small amount of tissue [23,24,25]. As in the case of other species [26,27,28,29], sets of microsatellite markers for the root vole have been developed and tested [23,30,31,32,33,34,35].

The aim of this paper was to assess the effects of the decline and fragmentation of wetland habitats on the genetic structure of the root vole population. Using microsatellites as molecular markers, we expected to define genetically distinct subpopulations on a local scale, determine the distances between patches of wetlands that restrict gene flow, and indicate the main barriers (natural or anthropogenic) hindering the migration of the studied species.

## 2. Materials and Methods

### 2.1. Study Area and Tissue Sampling

Samples were collected in the area of Kampinoski National Park (coordinates: 52°19′ N 20°34′ E) situated in central Poland near Warsaw. The park covers an area of 385.44 km^2^. There is a mosaic of forest and non-forest habitats in this area, and wet habitats (required for the root vole) are separated by contrasting dry habitats formed on belts of postglacial sand dunes covered mainly by pine forests (*Peucedano-Pinetum* and *Leucobryo-Pinetum*). In addition, there are anthropogenic elements (cultivated fields, roads, and buildings), but their share is small in this area. 

The habitat changes and the basic abiotic parameters of this area have been documented over the last few decades, especially since the National Park was established in 1959 [36]. One of the most important changes was the lowering of the groundwater level, which resulted in many transformations in the nature of the park, including a complete disappearance of some surface waters. Moreover, over the past decades, the share of forested land has increased significantly: from 18,600 hectares in 1956 to 26,700 hectares at the beginning of the 21st century. As a result of these changes, the root vole significantly decreased its range in the park in the second half of the 20th century [36], but the genetic consequences of these transformations have not been studied.

**Figure 1:**
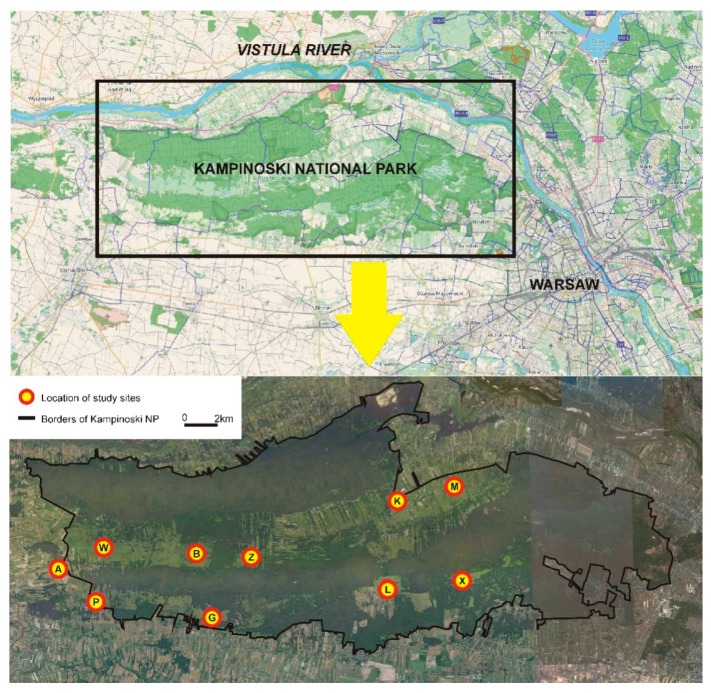
Study area (Kampinoski National Park) and the schematic location of sites where rodents were caught and tissue material for genetic testing was collected. The study sites lie in two swamp belts (northern and southern, light green) separated by a belt of glacial dunes covered by pine forests. Northern swampy belt—study sites W, B, Z, K, and M; southern swampy belt—study sites A, P, G, L, and X.

Samples were collected from 10 sites: 5 from the northern swampy belt and 5 from the southern swampy belt. Two sampling sites in the western and eastern part and one site in the central part of each swampy belt were chosen (Figure 1). The distance between the sampling sites (subpopulations) ranged from 2.9 km to 25.4 km, with an average distance (calculated on the basis of all possible combinations between sites) of 12.2 km. 

The northern swampy belt with the W, B, Z, K, and M study sites is bordered by wide dune belts covered by coniferous forest in the north and south. It adjoins the Vistula River valley and an urbanised area in the east and reaches an agricultural area and the Bzura River valley in the west. The southern swampy belt with the A, P, G, L, and X study sites is bordered by a dune belt with a coniferous forest in the north and by a forest and infrastructure of the city of Warsaw in the east. The other parts of the belt adjoin agricultural areas and scattered buildings. In the southern swampy belt, the relative continuity of sedge habitats is maintained only in the western part, and there are several small and isolated open areas covered by sedges in the central and eastern part. 

### 2.2. Small Mammal Trapping

Root voles were captured in patches of sedge communities. A transect of 50 wooden live traps spaced at 20-m intervals was set within each patch. The trapping lasted for 2–4 consecutive days and all captured rodents were identified to the species, sexed, and weighed. When the rodent was identified as a root vole, a tissue sample for DNA analysis was taken by toe clipping (permission for this procedure was obtained from the ethics committee). The clipped toes were transferred into sterile Eppendorf tubes and dried using silica gel. Captured individuals were released in the site of capture. Trapping sessions were carried out in the autumn (from October to November) of 2008 and 2009. Based on previous studies [31], it is known that the population of the root vole, even when undergoing significant demographic and social changes (in terms of the density and kin structure), may maintain a high genetic variability and a stable genetic composition between years. In the case of our study, there were no differences in the success of trapping the root vole between the years (population density was similar); therefore, all collected tissue samples were summed up and the factor “study year” was not included in the analysis (Table 1).

### 2.3. Microsatellite Genotyping

DNA from dried toes was isolated with chelex resin pursuant to the protocol proposed by Larbi [37]. The concentration of isolated DNA was measured with a Nanodrop 2000 Spectrophotometer (Thermo Scientific, Waltham, MA, USA). The variability of the DNA of 218 individuals was assessed by genotyping 9 microsatellite loci: Ma35, Ma54, Ma66, Ma88, MAG6, MSMM2, MSMM5, MSMM6, and MSMM7 [38]. A multiplex-touchdown PCR with 9 primer pairs labelled with PET, FAM, NED, and VIC fluorescent dyes [38] was performed. The PCR was conducted in a T-Gradient thermocycler (Biometra, Göttingen, Germany). The volume of each amplified sample was 8.0 μL and consisted of 1.0 μL of DNA (concentration between 10 and 20 ng/μL), 4.0 μL of AmpliTaq Gold 360 Master Mix (Life Technologies, Carlsbad, CA, USA), a primer set (from 0.06 μL to 0.15 μL), and sterile MilliQ water (Millipore). The PCR steps were programmed as follows: denaturation: 94 °C (10 min); hybridisation 60 °C to 56 °C (30 s); annealing 72 °C (90 s); 35 cycles. The capillary electrophoresis of the PCR products was carried out in an ABI3500 Genetic Analyzer (Life Technologies), and the genotypes were analysed in GeneMapper v4.1 against the internal LIZ 600 size standard. A total of 15% of the sample was rerun to clarify ambiguous signals and to ensure the precision of the genotyping through duplication.

### 2.4. Analyses of Genetic Structure and Variability

Standard genetic diversity indices were estimated with Genodive v. 3.02 [39] and FSTAT v. 2.9.3.2 [38]. We calculated the number of alleles (N_a_), allelic richness (A_R_), observed (H_o_) and expected (H_e_) heterozygosity, and the inbreeding coefficient (Gis) for all subpopulations. Pairwise differentiation based on G’st [40] between ten subpopulations was performed using GenoDive.

Deviations from the Hardy–Weinberg equilibrium (HWE) were examined for each population calculating the inbreeding coefficient (Gis) with GenoDive and GenAlEx v. 6.5 [41]. The significance of each pair of sites with 9999 permutations was tested. Additionally, the deviation of subpopulations from HWE was tested with exact tests assessing heterozygote deficiency in GenePop v 4.7 [42,43]. We applied a Bonferroni correction for multiple comparisons. The frequency of null alleles and linkage disequilibrium was estimated in FSTAT and GenePop. Probability of Identity for unrelated (PI) and related (PI sibs) individuals was calculated in GenAlEx. 

To test the proportion of genetic variance between individuals and subpopulations, we performed an Analysis of Molecular Variance (AMOVA) for standardized data [44] implemented in GenAlEx. Statistical significance was assessed with 9999 permutations. A Principal Coordinates Analysis (PCoA) based on G’st genetic distance between subpopulations was performed in GenAlEx [44]. Isolation by distance (IBD) was calculated with the use of the Mantel test with GenAlEx using 9999 permutations. The genetic distance was expressed as G_st_/(1 − G_st_) between the groups. A genetic tree based on G’st matrices [45] and an UPMGA (unweight pair-group method with arithmetic mean) algorithm [46] was constructed using Mega-X [47].

The Bayesian clustering method implemented in Structure v. 2.3.4 [48] to determine the number of clusters (K) based on the prior information on the population was applied. To illustrate the genetic structure of the population, 20 independent runs with K = 1 to 10 were carried out with 10^6^ Markov chain Monte Carlo (MCMC) iterations and a burn-in period of 10^5^. Admixture and correlation models were implemented. Structure Harvester was used to assess the optimal value of K for this study (inspection of log-likelihood values and according to the ∆K method developed by Evanno [49]). The raw Structure output files were combined and visualised using Clumpak v. 1.1 [50].

## 3. Results

The number of alleles per locus ranged from 8 (MM5) to 13 (Ma54), with an average of 9.78. The average allelic richness and effective number of alleles was 6.47 and 4.16, respectively. Allelic richness (AR) across subpopulations ranged from 5.40 to 6.47, with the highest in deme B and the lowest in deme K. Two private alleles were found: in deme M (Ma66—allele 282) and in deme P (Ma54‚ allele 239). We found a significant heterozygosity deficit in 8 out of 9 loci (with the Bonferroni correction for multiple tests). Due to a lack of evidence for null alleles or linkage disequilibrium, all loci were kept for further analyses. The PI analysis showed that using two markers (for unrelated individuals) and fiver markers (for related individuals) reduces the likelihood of finding two individuals with the same genotype for the certain loci below 1% (0.02% and 0.6%, respectively). Table 2 shows the genetic diversity of ten root vole subpopulations based on nine microsatellite loci. The observed heterozygosity was significantly lower than expected in all sites which indicates heterozygote deficiency. This was confirmed by significant inbreeding coefficient (Gis). 

**Figure 2:**
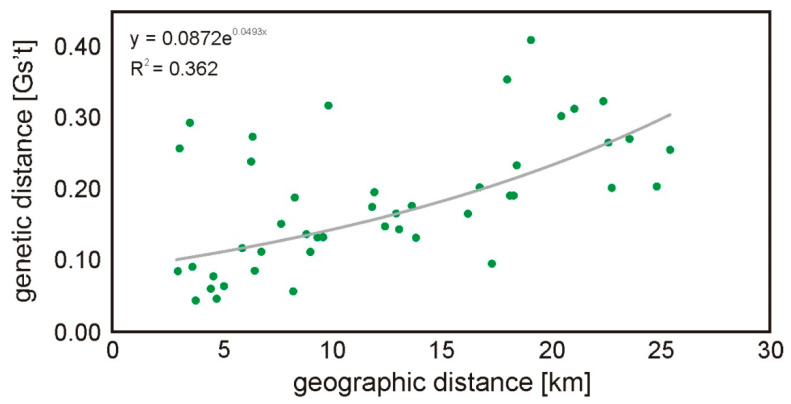
Regression between the genetic and geographical distance in km among all root vole groups (Mantel test of correlation, r_xy_ = 0.568; *p* = 0.003). Each dot represents a comparison between two sites.

All subpopulations were included in pairwise G’st analyses and the overall G’st was 0.042 (SD 0.007; 95% CI: 0.029–0.053). The highest G’st values were observed for P and K (0.111; *p*-value < 0.001). Pairwise genetic differentiation was significant in most of the comparisons, except in pairs B-Z and G-Z (Table 3). The Mantel test indicated a significant positive correlation (r_xy_ = 0.568; *p* = 0.003; Figure 2) between the geographic and genetic distances.

**Figure 3:**
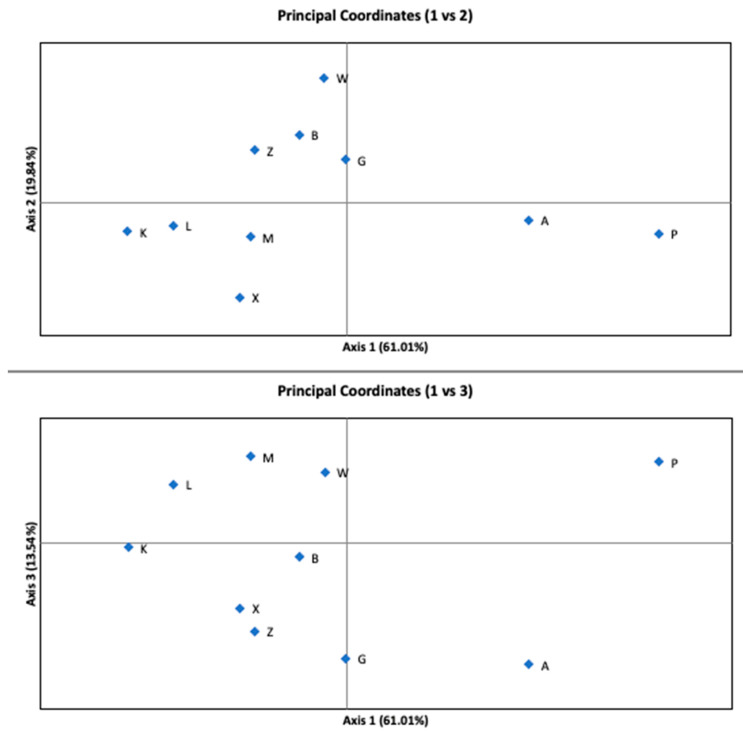
Principal Coordinates Analysis (PCoA) performed for ten subpopulations showing the 1st vs. 2nd and the 1st vs. 3rd axes. The explained variance is written in parentheses on each axis.

The AMOVA indicated that the variance among subpopulations accounted for only 4% (*p*-value < 0.001) of the total variance. Most of the variability occurred within the sites. Figure 3 shows the results of the PCoA analysis of G’st distance. Coordinate 1 (axis 1) explains 61.01%, coordinate 2 (axis 2) explains 19.84%, and coordinate 3 (axis 3) explains 13.54% of the variation. The cumulative percentage of the variation explained by PCoA was 94.38%. Subpopulations A and P were separated by the first axis. 

**Figure 4:**
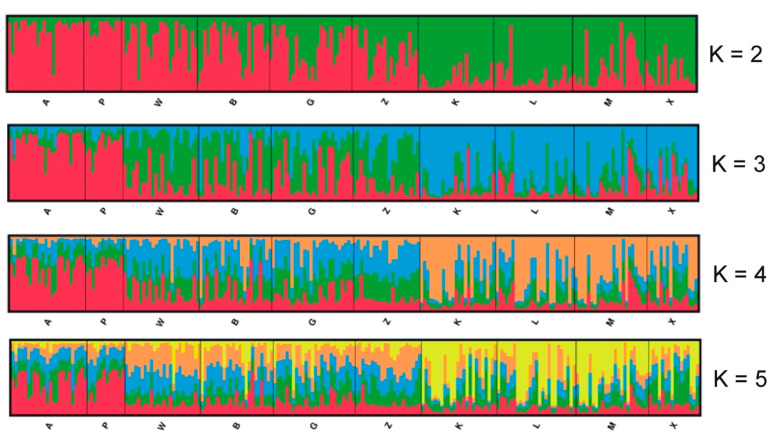
Results of the Bayesian clustering of 218 root voles from 10 sites in the Kampinoski National Park. The Structure plots represent separate runs when K was fixed at 2–5. Individuals are ordered by the location and each of them are represented by the line proportionally divided into colour segments. Black lines separate the individuals from different subpopulations.

**Figure 5:**
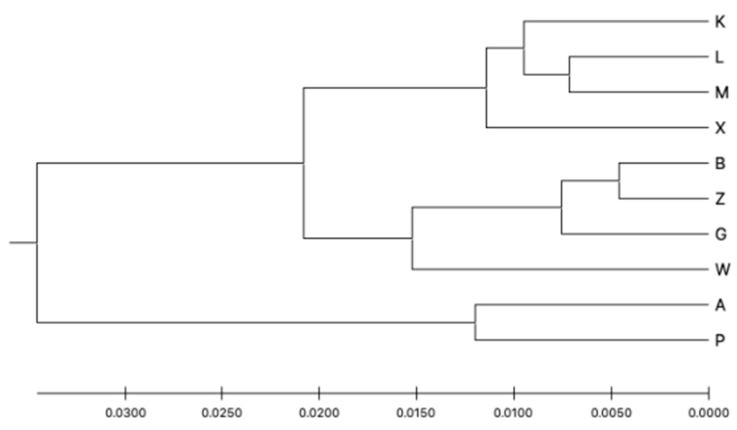
Genetic distance UPGMA tree based on the G’st genetic distance between the subpopulations.

Structure Harvester showed that the optimal number of clusters by the Evanno method was K = 2 and the second most likely was K = 5. The Structure analysis is presented in Figure 4. It seems that the capture site did not affect the group assignment. The dendrogram based on the UPGMA method calculated using the G’st genetic distance (Figure 5) also divided the entire population into two groups. The first group could also be split into two parts consisting of sites K, L, M, X and B, Z, W, and G. The second group included sites A and P. 

**Figure 6:**
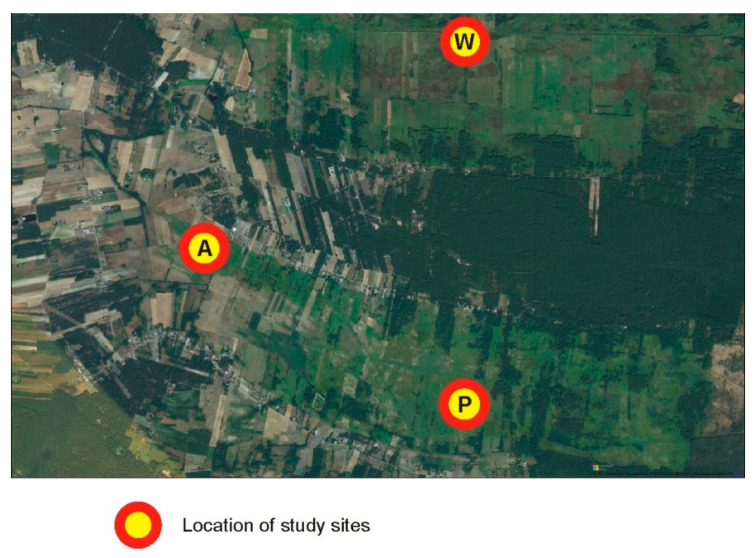
Enlarged eastern part of the study area where subpopulations A and P were separated from subpopulation W by a human settlement, which appears to be an effective barrier for gene flow.

Groups A and P were separated from W by a human settlement, which appears to be an effective barrier (Figure 6), compared to the suboptimal habitats within the belt.

## 4. Discussion

In this study, we focused on the decline and fragmentation of wetland habitats and studied this problem on a local scale for a habitat specialist—the root vole. We expected that the natural habitat barriers and the progressive decline in the habitats would affect the genetic parameters of the root vole. We also expected to observe an impact of two types of isolation: one caused by the dry forested dune belts separating the marsh strips and the other one caused by the fragmentation of the non-forest wetland habitats due to the multiyear (since the 1950s) decline in the groundwater level and the increasing forest cover (described in detail in [36]). In general, however, we found that despite the fragmentation, the studied population turned out to be genetically similar, and the main factor differentiating the populations was the isolation by distance. The geographic distance however explained only 36% of the genetic distance variability, and other factors were not analyzed. Previous studies of root vole populations in eastern Poland (Narewka River valley in Białowieża Forest) conducted by the authors of [31] also showed a heterozygosity deficit even in years with high densities, but this open cyclic population generally had a high level of genetic variability.

In contrast to our predictions, the main division of the vole population in the study area did not run along the north–south line (between the two swamp belts) but along the east–west line. This means that the belts of dry glacial dunes covered by pine forest do not constitute a significant barrier to gene exchange between populations. It should be noted here that the differences in the relative height between the tops of the dunes and the almost flat areas of the swamp belts are up to 30 m, and the width of these belts is usually more than two kilometres. Therefore, it was assumed that migration, at least theoretically, should be limited not only by the unfavourable habitat (dry pine forest) but also by the topography of the area. 

The ecological literature provides no data indicating natural terrestrial ecosystems that may constitute significant barriers to the migration of voles. Kelemen et al. [23] showed that the populations of the Pannonian root vole in the fragmented habitats of an agricultural landscape of Slovakia did not show isolation by distance, but their studies were carried out on a small spatial scale and there was a channel in the study area—a potential migration corridor connecting the studied populations. Data from Lithuania, where the root vole has expanded its distribution range, show that this species can be caught even in dry meadows, wastelands, and even commercial fruit farms and that abandoned agricultural areas promote its wider distribution [51,52]. Moreover, as reported by the authors of [53], the root vole was captured in atypical dry habitats (xerothermic grassland), probably because this species is common in the neighbouring wet habitats. Such a result may indicate that, although the species is considered a habitat specialist [20,54], the root vole has high migratory abilities and, after losing the continuity of wet habitats, can still effectively implement gene exchange between subpopulations crossing suboptimal and unsuitable habitats. This agrees with the results reported by the authors of [55], where the relatively frequent occurrence of the root vole in the area of the Kampinoski National Park from 1980 to 2012 was shown. However, large natural barriers, including wide rivers and anthropogenic factors, can be a significant barrier to migration [21,56,57,58,59,60]. For example, the authors of [22] found a significant level of differentiation between local populations of the root vole located 5–15 km apart and separated by anthropogenic barriers—dykes. This indicates that an anthropogenic dyke with a relatively small height and width isolates populations much more effectively than a dune that is several kilometres wide, several dozen meters high, and is additionally covered by dry forest. 

The isolating effect of anthropogenic barriers on the genetic structure of the root voles cannot be unequivocally demonstrated, because the Kampinoski National Park has only a few elements of this type. Only one large asphalt road (7 m wide; provincial road number 579) runs through the park, with an average traffic of about 6000 vehicles per day. This road divides the park into the eastern and western parts, and a similar division was exhibited by the genetic structure of the studied vole population. It has been emphasised in many studies that roads can be perceived as a barrier to rodent movement [61,62,63,64,65,66]. However, this convergence does not justify the claim that this road is the main barrier. The genetic differences observed between the eastern and western subpopulations may also be a result of the complementation of the root vole population in the Kampinoski National Park by migrants from two different directions. Probably, the eastern subpopulation can maintain the flow of individuals with the Vistula River valley (about 7 km to the northeast from subpopulation M) and the western subpopulation—with the Bzura River valley (about 6 km to the west from subpopulation W). All these populations retain genetic similarity, but they have developed measurable differentiation due to the distance of separation. It cannot be concluded based on the analysed data that the road crossing the park is the direct cause of the genetic division of the vole population.

Rural buildings are another type of a potential anthropogenic barrier in the park. Our research has shown that subpopulations A and P show clear genetic differences from subpopulation W, and these differences cannot be explained by geographic distance. These subpopulations are divided by a line of rural buildings, but the infrastructure (buildings, fences, paved surfaces) is scattered and does not constitute a tight barrier. The role of the barrier effect of such rural infrastructure can be reinforced by companion animals, i.e., cats and dogs, which can prey on voles migrating through the building line [67,68]. Can the presence of such infrastructure be an effective barrier to the movement of voles? We cannot clearly prejudge this, as such an effect occurred only in one part of the studied area. We do not have a comparable control area, because the rural buildings in the park are sparse and scattered. Interpretation doubts are also raised by the fact that subpopulations A and P also differ from subpopulations G. Although population G lies further than W, it is not separated by a barrier in the form of building lines, and we should expect greater genetic similarity. Taking into account the specificity of the studied area (a small number of anthropogenic elements), we are not able to conclude from the present results that such elements significantly affect the genetic structure of the studied voles. However, we must note that the strongest effects were observed in sites where the influence of anthropogenic barriers is at least probable. This issue therefore requires more focused research.

The above-described ability of the root vole to overcome even wide and unfavourable terrestrial natural barriers certainly does not mean that the drying out of the park’s wetlands will not have any negative consequences for this species. Changes in the surface of the wet habitats and their fragmentation will certainly affect the range and abundance of the species, which was previously pointed out by the author of [36]. Habitat patchiness has been found to change the processes of dispersal of individuals and thereby increase the aggregation of relatives and individuals in general [69]. In addition, seasonal fluctuations in the size of fragmented populations (theoretically more susceptible to drift) may also affect the genetic structure of this population [31]. The genetic effects of cyclical or progressive wetland drying and the fragmentation of the vole population, however, may be compensated (delayed) by the species’ migratory capacity. This supposition is consistent with one of the fundamental principles of genetics, which says that the loss of genetic diversity due to drift may be counterbalanced by immigration [70]. Obviously, achievement of the same type of balance between gene flow and genetic drift is influenced by a whole range of population factors such as the population size, temporal variation, type of mating, kin structure, and dispersal patterns [31,71,72,73,74,75]. A number of other factors should therefore be examined in order to determine a spatial arrangement of wet habitats that ensures the sustainable functioning of root voles and their high heterozygosity and genetic variability.

## 5. Conclusions

Despite the spatial fragmentation, the studied population turned out to be poorly genetically diverse, and the isolation through distance was the main differentiating factor. Natural habitats and topography (even wide belts of dry glacial dunes up to 30 m height covered by pine forest) did not constitute a significant barrier to gene exchange between root vole subpopulations. As a result, the genetic effects of the fragmentation of wetlands in the Kampinoski National Park were substantially compensated by the migratory abilities of this species. Our study does not provide clear results on the impact of anthropogenic barriers but suggests that such barriers may have a much stronger effect than natural barriers. 

## Figures and Tables

**Table 1:** Number of samples analysed for each study site.

		WEST		CENTRE	EAST	
**Northern Strip**	***Study site***	**W**	**B**	Z	K	M
	*Number of samples*	24	23	21	24	23
**Southern Strip**	*Study site*	A	P	G	L	X
	*Number of samples*	24	12	26	25	16

**Table 2:** Genetic diversity of ten root vole subpopulations in the Kampinoski National Park based on 9 microsatellite loci.

Location	Na	AR	Ho	He	HWE		Gis
					*p*-Value	±SE	
A	6.89	5.53	0.446	0.751	0.000	0.000	0.406 ***
P	6.22	5.89	0.437	0.755	0.000	0.000	0.422 ***
W	7.56	6.00	0.656	0.781	0.000	0.000	0.160 ***
B	8.44	6.47	0.662	0.806	0.000	0.000	0.178 ***
G	7.44	6.04	0.677	0.792	0.000	0.000	0.146 ***
Z	6.89	6.09	0.564	0.822	0.000	0.000	0.314 ***
K	6.44	5.40	0.628	0.749	0.000	0.000	0.162 ***
L	6.89	5.51	0.703	0.771	0.011	0.007	0.088 **
M	7.56	5.94	0.695	0.780	0.000	0.000	0.108 **
X	6.33	5.40	0.513	0.762	0.000	0.000	0.326 ***

Na—mean number of alleles; AR—allelic richness; Ho—observed heterozygosity; He—expected heterozygosity; HWE—*p*-values and standard errors of Hardy–Weinberg equilibrium exact tests (with Bonferroni correction); Gis—inbreeding coefficient (** *p* < 0.01; *** *p* < 0.001);

**Table 3:** Pairwise differentiation between ten root vole subpopulations in the Kampinoski National Park. Below diagonal—values of G’st. Above diagonal—*p*-values of G’st implemented in GinoDive.

	A	B	G	K	L	M	P	W	X	Z
A	--	0.000	0.000	0.000	0.000	0.000	0.036	0.000	0.000	0.000
B	0.046	--	0.002	0.000	0.000	0.003	0.000	0.000	0.000	0.082
G	0.029	0.020	--	0.000	0.000	0.000	0.000	0.000	0.000	0.064
K	0.086	0.041	0.045	--	0.006	0.004	0.000	0.000	0.007	0.000
L	0.080	0.035	0.042	0.017	--	0.017	0.000	0.000	0.034	0.000
M	0.066	0.022	0.045	0.021	0.014	--	0.000	0.000	0.005	0.001
P	0.024	0.066	0.060	0.111	0.092	0.069	--	0.000	0.000	0.000
W	0.066	0.027	0.036	0.060	0.048	0.049	0.075	--	0.000	0.000
X	0.055	0.048	0.041	0.024	0.016	0.029	0.086	0.067	--	0.001
Z	0.046	0.009	0.010	0.031	0.030	0.029	0.073	0.029	0.033	--

## Data Availability

Not applicable.
